# Transporting Lung Cancer Screening Effects From National Lung Screening Trial to a US Screening-Eligible Population

**DOI:** 10.1200/CCI-25-00280

**Published:** 2026-10-26

**Authors:** I-Hsuan Su, Louise M. Henderson, Xiaomeng Chen, Michael G. Hudgens, Daniel S. Reuland, M. Patricia Rivera, Courtney Schlusser, Til Stürmer, Michael Webster-Clark, Jennifer L. Lund

**Affiliations:** ^1^ Department of Epidemiology, University of North Carolina at Chapel Hill, Chapel Hill, NC; ^2^ Lineberger Comprehensive Cancer Center, University of North Carolina at Chapel Hill, Chapel Hill, NC; ^3^ Department of Radiology, School of Medicine, University of North Carolina at Chapel Hill, Chapel Hill, NC; ^4^ Department of Biostatistics, University of North Carolina at Chapel Hill, Chapel Hill, NC; ^5^ Division of General Medicine and Clinical Epidemiology, School of Medicine, University of North Carolina at Chapel Hill, Chapel Hill, NC; ^6^ Division of Pulmonary and Critical Care Medicine, Department of Medicine, University of Rochester, New York, NY; ^7^ Division of Public Health Sciences, Wake Forest University School of Medicine, Winston-Salem, NC

## Abstract

**Purpose:**

The National Lung Screening Trial (NLST) found that screening with annual low-dose computed tomography (LDCT) reduces lung cancer (LC) mortality. Because studies have shown that the US population eligible for screening differs from NLST, we sought to quantitatively extend the NLST findings to this population.

**Methods:**

We reweighted individual-level NLST data using inverse odds weights (IOW) to the covariate distribution of a contemporary screening-eligible population from the 2022 Behavioral Risk Factor Surveillance System (BRFSS) data. In the IOW and unweighted NLST population, we estimated the LC-specific and all-cause mortality among individuals undergoing three annual screenings of LDCT compared with chest radiography, as well as false-positive rates in the LDCT arm only.

**Results:**

We included 53,454 NLST participants and 7,265,314 survey-weighted screening-eligible BRFSS respondents (unweighted sample of 15,779). The BRFSS population was older, more racially and ethnically diverse, and more likely to currently smoke and have respiratory comorbidities. We estimated similar benefits of screening on LC and all-cause mortality in the weighted versus unweighted NLST population (5-year LC risk difference [RD; 95% CI], –29 [–54, 4] *v* –23 [–44, 3]; all-cause RD [95% CI], –44 [–95, 8] *v* –37 [–66, –7] deaths per 10,000 screened). Estimated false-positive rates were higher in the weighted versus unweighted NLST population across three annual screenings.

**Conclusion:**

We estimated similar mortality benefits and slightly increased false-positive rates from LDCT in the weighted versus unweighted NLST population. Our findings account for the potential differences within subgroups of interest but not variations in longitudinal adherence and diagnostic work-up between trial and clinical practice.

## Introduction

Lung cancer (LC) is the leading cause of cancer-related death in the United States.^1^ According to the American Cancer Society, around 229,410 new cases of LC and 124,990 deaths from LC will occur in 2026.^2^ Stage at LC diagnosis is strongly correlated with survival, with 5-year survival rates of 67% in local disease (stages I and II) and 12% in distant disease (stages III and IV) for non–small cell LC.^3^ As prognosis is poor for later-stage disease, facilitation of early detection of LC is imperative.

**Context**

**Key Objective**
What would have been the effect of screening with low-dose computed tomography (LDCT) if the National Lung Screening Trial (NLST) had been conducted in a population more aligned with those eligible for screening in real-world clinical practice?
**Knowledge Generated**
We found similar and potentially slightly greater benefits of screening with LDCT versus chest radiography between NLST and the real-world screening-eligible population in reducing lung cancer (LC) and all-cause mortality. However, false-positive rates of screening were higher in the population weighted to reflect real-world practice.
**Relevance *(M. Behera)***
This study provides important evidence that the mortality benefits of LDCT LC screening observed in the NLST are likely to be applicable to today's US screening-eligible population, despite substantial demographic and clinical differences between trial participants and real-world patients.*
*Relevance section written by *JCO* *Clinical Cancer Informatics* Associate Editor Madhusmita Behera, PhD.

The National Lung Screening Trial (NLST) is the largest trial to assess the effects of LC screening by low-dose computed tomography (LDCT) and found a 20% relative reduction in LC mortality among those who currently smoke or formerly smoked who received three annual screenings of LDCT compared with chest radiography (CXR).^4^ The trial also found that 96.4% of all positive screens in the LDCT group and 94.5% of all positive screens in the CXR group were false positives.^4^

Following results from the NLST, the US Preventive Services Task Force (USPSTF) issued a grade B recommendation in 2013 for annual LC screening (LCS) with LDCT and further updated it in 2021 to include adults age 50-80 years who have at least 20 pack-years of smoking history and currently smoking or have quit within the past 15 years.^5,6^

However, studies have reported differences in the distribution of characteristics between the NLST and the US population eligible for LCS.^7,8^ As factors such as age, gender, race and ethnicity, smoking status, and comorbidities are associated with LC, LC mortality, and all-cause mortality, these factors may modify the benefits and harms of LCS and in turn lead to concerns about the generalizability of the NLST findings to real-world clinical practice.^9-11^ Therefore, more realistic estimates that reflect the expected effects of screening in the real-world eligible population are necessary to inform policy and clinical guidelines. Furthermore, it is important to consider both the benefits of mortality reduction and the harms associated with false-positive findings when assessing the effects of LCS at the population level.

Recently, quantitative methods have been developed for transporting (or generalizing) estimated treatment effects from trials to target populations to enhance the evidence base for population-level decision making.^12-15^ Prior studies by Inoue et al and Prosper et al used inverse odds weighting to estimate the treatment effects of screening with LDCT in simulations.^16,17^ Although a study conducted by Robertson et al transported NLST benefits to a target population based on the 2010 National Health Interview Survey,^18^ we sought to transport the inferences of NLST to a more contemporaneous screening-eligible US population derived from the 2022 Behavioral Risk Factor Surveillance System (BRFSS) data, including both measures of benefit (eg, reductions in LC-specific mortality) and harm (ie, increases in false-positive rates), providing a more comprehensive view of the net benefits of LC screening.

## Methods

The institutional review board of the University of North Carolina at Chapel Hill determined that this study was not human subjects research and was exempt from review (24-0772).

### Data Source, Study Population, and Study Design

#### Randomized Controlled Trial Population

NLST was a multicenter, randomized controlled trial to compare the effect of LCS with LDCT versus CXR on outcomes of LC mortality.^19^ The trial enrolled 53,454 individuals from August 2002 through April 2004.^4^ Eligible participants were between 55 and 74 years of age at the time of random assignment, had a history of cigarette smoking of at least 30 pack-years, and, if formerly smoked, had quit within the previous 15 years.^4^ There were 26,722 participants randomly assigned to three annual rounds of screening with LDCT and 26,732 to screening with CXR.

NLST data are readily available to researchers following approval of a proposed project by the National Cancer Institute.^20^ However, data available to researchers do not align perfectly with the original trial paper even after applying adjustments to the data according to NCI recommendations.^4^ Details on data adjustments and the comparability of our results are discussed in Supplementary Material Section 1, Tables S1 and S2.

#### Real-World Screening-Eligible Population

The screening-eligible population was derived using individual-level data from the BRFSS Core Module 2022. BRFSS is a cross-sectional, national, phone-based survey conducted by the Centers for Disease Control and Prevention on US residents regarding their health-related risk behaviors, chronic health conditions, and use of preventive services.^21^ In 2022, BRFSS collected data in all states and territories and completes more than 400,000 adult interviews each year.^21,22^ We decided to use the 2022 BRFSS data to define our real-world population as the LCS module is part of the core module.

We mirrored the NLST eligibility criteria when defining the BRFSS screening-eligible population. We detailed the process of applying the NLST eligibility criteria to BRFSS respondents and the identifiability criteria for transport analysis in Supplementary Material Section 2, Figure S1 and Section 3.

### Transportability Analysis

We aimed to extend the results from NLST to the BRFSS 2022 screening-eligible population using inverse odds weights (IOW). This method involved estimating the odds of trial participation through logistic regression, combining data from both NLST and the BRFSS screening-eligible population, with trial participation as the outcome and potential effect measure modifiers (EMMs) as predictors.^23,24^ EMMs are variables that are both predictors of the outcome and differ in their distribution between NLST and the screening-eligible population.^23,25^ The NLST population was then weighted by the IOW to create a weighted trial population with the distribution of effect measure modifiers reflecting that of the BRFSS screening-eligible population.^23,24^ Finally, we estimated the outcomes of interest using this weighted NLST population.^23,24^

#### Exposure Assessment

Our exposure is random assignment to three rounds of annual LDCT or CXR screening, as recorded in the NLST data.

#### Outcome Assessment

Our primary benefit end points were LC-specific and all-cause mortality determined by the NLST team.^4^ Our secondary benefit end point was the percentage of all diagnosed LC that was early-stage at the time of diagnosis. We used the American Joint Committee on Cancer (AJCC) 7th edition staging and categorized stages I and II as early-stage.

Our primary harm end point was false-positive rate. To reflect current clinical practice, we determined positive screens using the Lung Imaging Reporting and Data System (Lung-RADS) criteria that were retrospectively applied to the LDCT arm of the original NLST data.^26^ We identified screens with a Lung-RADS category 3 and 4 as positive. Since Lung-RADS was not applied to the CXR arm, these analyses were limited to the LCDT arm alone.

The false-positive rate is the number of false-positive screens divided by the sum of false-positive and true-negative screens as measured in prior research.^26^ We define a false-positive screen as a positive Lung-RADS screen that did not result in an LC diagnosis within a year and a true-negative screen as a negative Lung-RADS screen that did not result in an LC diagnosis within a year. We also estimated the percentages of false-positive screens among all positive screens as a secondary analysis to be compared with the original NLST paper.^4^ Detailed definitions of these harm outcomes can be found in Supplementary Material Section 4, Table S3.

#### Covariate Assessment

We considered the following variables as potential EMMs based on literature and clinical input: age, sex, race, ethnicity, body mass index, pack-years of smoking, years since smoking cessation, and presence of emphysema, chronic obstructive pulmonary disease, or chronic bronchitis (referred to as the presence of respiratory condition).^27^ We scaled age and BMI by a factor of 5, and pack-years by a factor of 20, to obtain units in increments that are more clinically meaningful and close to the standard deviation of the data to improve model fit and efficiency.

As the numbers of participants who were non-White compared with White were small in the trial population (8.5% non-White *v* 91.5% White; Table 1), we reclassified the race variable into White and non-White.

**TABLE 1.** Characteristics of the NLST and BRFSS Screening-Eligible Population

Characteristic	NLST, N = 53,452 No. (%)	BRFSS Screening-Eligible (survey sample–weighted)^a^, N = 7,265,314, No. (%)
Age category (years)^b^		
<50	2 (0.0)	NA
50-54	3 (0.0)	NA
55-59	22,860 (42.8)	1,738,645 (23.9)
60-64	16,369 (30.6)	2,302,867 (31.7)
65-69	9,517 (17.8)	1,799,859 (24.8)
70-74	4,697 (8.8)	1,423,943 (19.6)
75-79	4 (0.0)	NA
Age continuous (years)^b^		
Median (IQR)	60 (57, 65)	63 (59, 68)
Race^b^		
White	48,549 (91.5)	6,170,529 (87.7)
Black	2,376 (4.5)	532,513 (7.6)
Asian	1,095 (2.1)	901,64 (1.3)
Other	1,062 (2.0)	240,688 (3.4)
Missing	370	231,420
Ethnicity^b^		
Hispanic	935 (1.8)	407,990 (5.7)
Non-Hispanic	52,118 (98.2)	6,800,498 (94.3)
Missing	399	56,826
Sex		
Male	31,530 (59.0)	4,202,227 (57.8)
Female	21,922 (41.0)	3,063,087 (42.2)
Smoking status		
Current	25,760 (48.2)	4,052,602 (55.8)
Former	27,692 (51.8)	3,212,712 (44.2)
Pack-year category^b^		
<20	2 (0.00%)	NA
20-29	18 (0.02%)	NA
30-39	13,719 (21.78%)	1,967,800 (27.1)
40+	39,712 (78.20%)	5,297,514 (72.9)
Pack-year continuous^b^		
Median (IQR)	48 (39, 66)	46 (38, 56)
Years since smoking cessation^b^		
Current smoker	25,760 (48.5)	4,052,602 (55.8)
≤5 years	11,338 (21.3)	1,144,285 (15.8)
6-10 years	7,764 (14.6)	1,062,394 (14.6)
11-15 years	7,963 (15.0)	1,006,033 (13.9)
15+ years	307 (0.6)	NA
Missing	320 (0.6)	
Years since smoking cessation^b^		
Median (IQR)	7 (3, 11)	7 (4, 11)
BMI continuous^b^		
Median (IQR)	27.3 (24.4, 30.5)	27.98 (24.32, 32.28)
Education		
<High school	3,249 (6.1)	1,292,279 (17.8)
High School/GED	12,712 (23.8)	2,637,082 (36.4)
Some college	19,711 (36.9)	2,449,231 (33.8)
College grad	16,546 (31.0)	872,716 (11.0)
Missing	1,234	14,007
COPD or emphysema or chronic bronchitis^b^		
Yes	9,326 (17.6)	2,638,174 (36.6)
No	43,758 (82.4)	4,577,847 (63.4)
Missing	368	49,293

Abbreviations: BRFSS, Behavioral Risk Factor Surveillance System; COPD, chronic obstructive pulmonary disease; GED, general educational development; NA, not applicable; NLST, National Lung Screening Trial.

a We incorporated the BRFSS survey weights to reweigh the sample back to the general US population. BRFSS survey weights adjust for noncoverage, nonresponse, and demographic differences between those sampled by BRFSS and the total state population.^28^

b These numbers were recorded before truncation and imputation, which are detailed in Supplemental Material Section 5.

### Statistical Analysis

#### Data Cleaning

We accounted for extreme values in our data using truncation and conducted single imputation for missing values. A more detailed description of data cleaning can be found in Supplementary Material Section 5.

#### Descriptive Statistics

We described key demographic and clinical characteristics of the NLST and the BRFSS screening-eligible population by calculating medians and interquartile ranges for continuous variables and proportions for categorical variables.

#### Inverse Odds Weight Model

To compute IOWs, we ran a weighted logistic regression model (incorporating the BRFSS survey sampling weights^29^), including the potential EMMs listed previously. We specified age, BMI, and pack-years with restricted cubic splines with three knots at the 5th, 50th, and 95th percentile of the combined NLST and BRFSS screening-eligible population for flexibility. We also included interactions between race and ethnicity, race and sex, race and smoking status, race and presence of respiratory condition, ethnicity and smoking status, ethnicity and presence of respiratory condition, age and smoking status, age and presence of respiratory condition, and age and years since smoking cessation.^27^ As sensitivity analysis, we additionally restricted our primary model to EMMs identified based on the Variable Importance for Treatment Transport (VITT) plot and assessments of marginal and conditional EMMs.^30^ Details are included in Supplementary Material Section 6. More details on the sensitivity analysis model specification can be found in Supplementary Material Section 7, Figures S2-S6, Tables S4-S6.

To assess the balance of potential EMMs, we calculated the standardized mean differences (SMDs) and created a love plot to visualize the distribution of EMMs in the trial versus the target population.^31^ If the absolute value of the SMD is <0.10, it is often seen as adequate covariate balance.^32^ We also calculated the average standardized absolute mean difference (ASAMD) as a summary of the overall balance of our variables.

### *IOW-* *Weighted* *Benefit Outcomes*

We estimated the benefit of LCS with LDCT in decreasing LC-specific and all-cause mortality by computing the cumulative incidence of mortality and the mortality rates. To estimate cumulative incidences of LC mortality, we used the IOW-weighted Aalen-Johansen estimator accounting for death from other causes as competing event. We plotted the cumulative incidence of LC mortality in the LDCT and CXR groups. We also calculated the 5-year weighted risk difference and risk ratios. We followed the same procedure for estimating the cumulative incidence of all-cause mortality but used the IOW-weighted Kaplan-Meier estimator as there are no competing risks. To compare our results with the published NLST results, we also estimated the LC and all-cause mortality rates by using an IOW-weighted Poisson regression model. As a secondary analysis, we estimated the IOW-weighted percentages of early-stage LC diagnosis.

### *IOW-* *Weighted* *Harm Outcomes*

For potential harms of screening, we calculated the IOW-weighted false-positive rate and the IOW-weighted percentages of false-positive screens.

#### Confidence Interval Estimation

We obtained the 95% confidence intervals for all measures using nonparametric bootstrapping with 200 replicates to estimate the standard error. For rates, incidences, percentages, and ratios, we calculated the Wald confidence intervals on the natural log scale and then transformed them to ensure the estimates fall within a logical range.

## Results

### Study Population Characteristics

The analysis included 53,452 NLST trial participants and 15,779 screening-eligible BRFSS respondents (7,265,314 survey sample–weighted). Compared with the trial population, individuals in the screening-eligible population were older, more racially and ethnically diverse, more likely to currently smoke, and more likely to self-report having COPD, emphysema, or chronic bronchitis (Table 1). Characteristics of the unweighted screening-eligible BRFSS respondents can be found in Supplementary Material Section 8, Table S7.

### IOW Model Diagnostic Evaluation

Results of the model diagnostics are presented in Supplementary Material Section 9, Figures S7-S9.

### LC-Specific and All-Cause Mortality

We observed a higher cumulative incidence of LC mortality in both the LDCT and the CXR in the IOW-weighted compared with the unweighted NLST population (Table 2, Fig 1). Overall, the relative benefit of LDCT versus CXR in reducing LC mortality was similar between the weighted and unweighted populations, with a more pronounced absolute benefit in the weighted population (Table 2, Fig 1)

**TABLE 2.** Cumulative Incidence of Five-Year Lung Cancer and All-Cause Mortality in Deaths per 10,000 Screened in the Unweighted and IOW-Weighted NLST Population

Population	LDCT (%)	CXR (%)	Risk Difference (%)	Risk Ratio
Lung cancer mortality				
Unweighted	118 (110, 128)	142 (128, 156)	–23 (–44, –3)	0.84 (0.74, 0.94)
IOW-weighted	149 (135, 165)	178 (161, 197)	–29 (–54, –4)	0.84 (0.72, 0.98)
All-cause mortality				
Unweighted	477 (457, 497)	513 (492, 535)	–37 (–66, –7)	0.93 (0.87, 0.98)
IOW-weighted	640 (578, 709)	684 (647, 723)	–44 (–95, 8)	0.94 (0.87, 1.01)

Abbreviations: CXR, chest radiography; IOW, inverse odds weights; LDCT, low-dose computed tomography; NLST, National Lung Screening Trial.

**Fig 1.**
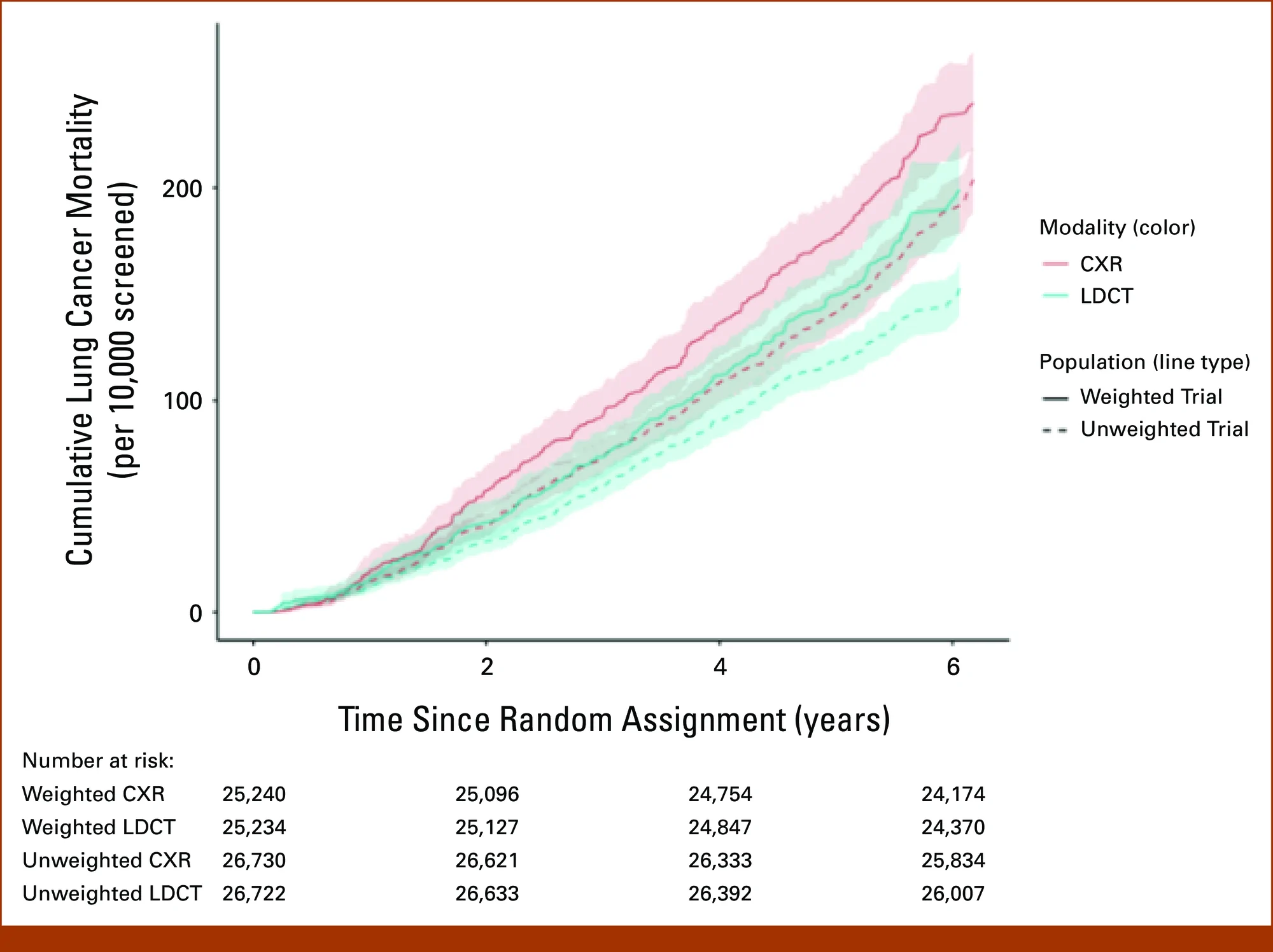
Cumulative incidence of lung cancer mortality in deaths per 10,000 screened for the LDCT (green) and chest radiography (red) arms in the IOW-weighted (solid line) and unweighted (dashed line) trial population. CXR, chest radiography; IOW, inverse odds weights; LDCT, low-dose computed tomography.

We observed similar trends in all-cause mortality, with higher incidences in both the LDCT and the CXR in the IOW-weighted compared with the unweighted trial population (Table 2, Fig 2). The relative benefit of LDCT compared with CXR on all-cause mortality was also similar between the IOW-weighted and unweighted NLST population with more pronounced relative benefit in the weighted NLST population (Table 2, Fig 2). Relatively similar patterns were seen in results from our sensitivity analyses with covariates based on the VITT plot and regression analysis (Supplementary Material Section 10, Table S8).

**Fig 2.**
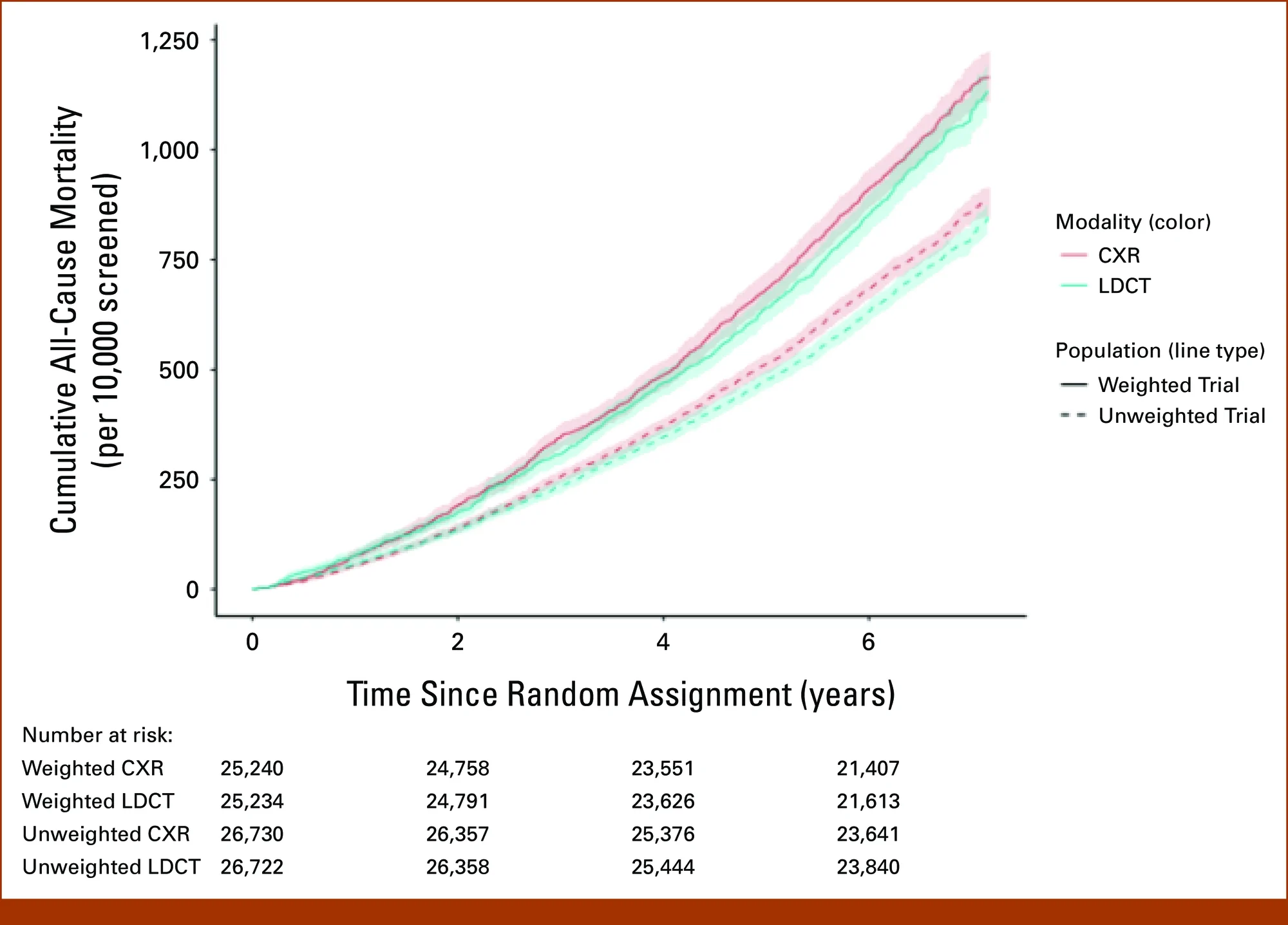
Cumulative incidence of all-cause mortality in deaths per 10,000 screened for the LDCT (green) and chest radiography (red) arms in the IOW-weighted (solid line) and unweighted (dashed line) trial population. CXR, chest radiography; IOW, inverse odds weights; LDCT, low-dose computed tomography.

### Mortality Rates

A similar, although slightly attenuated, absolute and relative benefit of LDCT versus CXR in reducing LC mortality was observed in the IOW-weighted population compared with the unweighted NLST population (Table 3). Elevated mortality rates were noted in both treatment arms in the IOW-weighted compared with the unweighted NLST population (Table 3).

**TABLE 3.** Lung Cancer and All-Cause Mortality Rates in Deaths per 100,000 Person-Years in the Unweighted and IOW-Weighted NLST Population

Population	LDCT	CXR	Rate Difference	Rate Ratio
Lung cancer mortality				
Unweighted	249 (230, 270)	312 (285, 342)	–63 (–106, –20)	0.80 (0.70, 0.91)
IOW-weighted	330 (298, 366)	390 (356, 427)	–60 (–111, –9)	0.83 (0.72, 0.96)
All-cause mortality				
Unweighted	1,137 (1,097, 1,178)	1,217 (1,178, 1,257)	–80 (–136, –23)	0.93 (0.89, 0.98)
IOW-weighted	1,550 (1,477, 1,627)	1,630 (1,558, 1,705)	–80 (–180, 19)	0.95 (0.89, 1.01)

Abbreviations: CXR, chest radiography; IOW, inverse odds weights; LDCT, low-dose computed tomography; NLST, National Lung Screening Trial.

There were similar absolute reduction and slightly attenuated relative reduction in all-cause mortality with LDCT versus CXR in the IOW-weighted compared with the unweighted NLST population (Table 3). Results from our sensitivity analyses are presented in Supplementary Material Section 10, Table S9.

### Percentage of Early-Stage LC Diagnosis

Similar to the unweighted trial population, a higher proportion of LCs were diagnosed at an early stage in the LDCT arm compared with the CXR arm across all screening rounds. Across the three screening rounds, 51.4%, 54.5%, and 56.3% of LCs were early-stage in the LDCT arm, compared with 35.8%, 37.0%, and 37.9% in the CXR arm (Supplementary Material Section 11, Table S10).

### False-Positive Results

In the LDCT arm, false-positive rates were higher in the IOW-weighted population compared with the unweighted NLST population across all three rounds of screening (Table 4). Results from our sensitivity analyses are presented in Supplementary Material Section 12, Table S11. We saw a similar pattern in false-positive rate findings in our sensitivity analysis. The percentage of false-positive screens was around 91% for both the unweighted and weighted NLST population (Supplementary Material Section 13 Table, S12).

**TABLE 4.** False-Positive Rate (%) of Screening Among LDCT Examinations Using Lung-RADS Criteria

Population	First Screen	Second Screen	Third Screen
Unweighted (LDCT only)	12.9 (12.6, 13.2)	5.4 (5.2, 5.6)	5.2 (5.0, 5.4)
IOW-weighted (LDCT only)	14.3 (13.9, 14.7)	6.2 (5.9, 6.5)	5.9 (5.6, 6.2)

Abbreviations: IOW, inverse odds weights; LDCT, low-dose computed tomography; RADS, Reporting and Data System.

## Discussion

We used a novel method of trial transport and diverse data sources to provide a more nuanced picture of the effects of LCS in a contemporary screening-eligible real-world population. We built upon prior transport studies by visualizing the cumulative benefits of screening on LC-specific and all-cause mortality and generating estimates of the harms, providing a more comprehensive picture of the net benefits of screening at the population level.

Similar to prior studies, NLST participants were younger, more likely to formerly smoke, be college educated, and less likely to report having serious respiratory conditions compared with our real-world screening-eligible population.^33,34^ Overall, we found that the relative and absolute measures of benefit were similar between the IOW-weighted and unweighted trial populations. We estimated a slight attenuation in the risk ratio and a modest increase in the risk difference when examining the 5-year incidence of mortality. Visualization of LC and all-cause mortality curves revealed an increased risk in both the LDCT and CXR arms for the IOW-weighted population compared with the unweighted NLST population, mainly due to older age, higher BMI, and higher prevalence of COPD in the screening-eligible population compared with the trial. However, the width of separation between the LDCT and CXR curves remained similar across both populations. Our mortality rate findings of similar benefits are also consistent with those recently reported by Robertson et al^18^ when comparing mortality rate ratios between NLST and a less contemporary target population characterized by the 2010 National Health Interview Survey.

We found that false-positive rates across three rounds of screening were 0.6%-2% higher in the IOW-weighted compared with the unweighted NLST population. False-positive findings and the resulting complications are more likely to occur in real-world clinical practice where the patient population tends to be older and has a higher prevalence of serious respiratory conditions. A recent study using a retrospective cohort also found higher false-positive rates of screening and downstream invasive procedures and complications in real-world clinical practice driven by differences in population characteristics and diagnostic management.^35^

Overall, our results indicate that the relative benefits of screening applied to a nationally representative, screening-eligible population are comparable with those observed in the NLST population, and absolute benefits might even be higher. This suggests that despite population differences in age and health conditions, we still see similar, if not slightly greater, benefits of LC screening among a real-world population, supporting the continuation of the USPSTF screening guidelines. On the other hand, higher false positives estimated among the weighted trial population, reflecting real-world practice, underscore the need to identify factors associated with false positives and for health systems to allocate resources focused on strategies to reduce false positives. Since false-positive findings may lead to unnecessary invasive procedures, focusing more resources on reducing false-positive findings through radiologist training, high-quality imaging technology, and informative shared decision making between patients and specialists can hopefully reduce the prevalence of invasive procedures and associated complications following a positive screen.

There are limitations that should be considered when interpreting our results. The trial transport method only addresses differences in baseline characteristics (potential EMMs) between the trial and screening-eligible real-world populations and thus assumes that adherence to screening recommendations, performance characteristics of screening test, and diagnostic work-up following screening in the real-world population is the same as in NLST. This is an implausible assumption, however, as NLST reported high adherence in the LDCT group at around 95%, and adherence in real-world clinical practice is low, with about 22%-60% of those who initiate screening returning for a second annual examination.^9,10,33,35,36^ Additionally, NLST participants were screened at academic instead of community medical facilities following a strict trial protocol. Thus, our estimates likely represent a ceiling for the true benefits that we would expect to accrue from LC screening in more heterogeneous clinical settings.^23^ We also assumed that we had accounted for all important effect measure modifiers of the relevant effects and correctly specified our trial sampling model. Thus, our estimates could be biased if either of these assumptions were violated.^23^ Some important effect measure modifiers that were not available in either the NLST or BRFSS data and could affect access to screening include socioeconomic status and health insurance coverage. Omitting these variables from our transport model might result in biased estimates. However, we found that our results were relatively similar across the different model specifications, which supports, to some extent, the robustness of our results against unmeasured covariates and model misspecification. Additionally, as clinical practices continue to evolve, using data from a historical trial and the 2022 BRFSS might not reflect contemporary clinical pattern.

In conclusion, the screening-eligible population represents a higher risk population with older age and higher levels of comorbidities. The benefits of LC screening on reducing LC and all-cause mortality are similar between NLST and the BRFSS screening-eligible population, whereas the expected harm of screening due to false-positive results is slightly higher in the screening-eligible population. All in all, our results add to the body of evidence supporting current screening guidelines by demonstrating screening benefits in a contemporary and representative screening-eligible US population. Future work should leverage alternative methods to additionally address the effects of differences in adherence to screening recommendations, performance characteristics of screening test, and diagnostic work-up following screening between the RCT and screening-eligible real-world populations.

## Supplementary Materials



## Data Availability

The data underlying this article are available through the National Cancer Institute Cancer Data Access System following project request approval at: https://cdas.cancer.gov/nlst/and the Centers for Disease Control Behavioral Risk Factor Surveillance System publicly available database at: https://www.cdc.gov/brfss/annual_data/annual_2022.html.

## References

[1] Siegel RL, Miller KD, Wagle NS, et al: Cancer statistics, 2023. CA Cancer J Clin 73:17-48, 2023 10.3322/caac.21763 36633525

[2] American Cancer Society: Key Statistics for Lung Cancer, 2026: https://www.cancer.org/cancer/types/lung-cancer/about/key-statistics.html

[3] American Cancer Society: Lung Cancer Survival Rates | 5-Year Survival Rates for Lung Cancer. https://www.cancer.org/cancer/types/lung-cancer/detection-diagnosis-staging/survival-rates.html

[4] National Lung Screening Trial Research Team, Aberle DR, Adams AM, et al: Reduced lung-cancer mortality with low-dose computed tomographic screening. N Engl J Med 365:395-409, 2011 10.1056/NEJMoa1102873 PMC4356534 21714641

[5] US Preventive Services Task Force, Krist AH, Davidson KW, et al: Screening for lung cancer: US preventive services task force recommendation statement. JAMA 325:962-970, 2021 10.1001/jama.2021.1117 33687470

[6] Moyer VA, U.S. Preventive Services Task Force: Screening for lung cancer: U.S. Preventive Services Task Force recommendation statement. Ann Intern Med 160:330-338, 2014 10.7326/M13-2771 24378917

[7] Howard DH, Richards TB, Bach PB, et al: Comorbidities, smoking status, and life expectancy among individuals eligible for lung cancer screening. Cancer 121:4341-4347, 2015 10.1002/cncr.29677 PMC4670572 26372542

[8] National Lung Screening Trial Research Team, Aberle DR, Adams AM, et al: Baseline characteristics of participants in the randomized national lung screening trial. J Natl Cancer Inst 102:1771-1779, 2010 10.1093/jnci/djq434 PMC2994863 21119104

[9] Lund JL, Rivera MP, Su IH, et al: Estimating the effects of cancer screening in clinical practice settings: The role of selective uptake and suboptimal adherence along the cancer screening continuum. Cancer Epidemiol Biomarkers Prev 33:984-988, 2024 10.1158/1055-9965.EPI-23-1491 PMC11351907 39012954

[10] Lopez-Olivo MA, Maki KG, Choi NJ, et al: Patient adherence to screening for lung cancer in the US: A systematic review and meta-analysis. JAMA Netw Open 3:e2025102, 2020 10.1001/jamanetworkopen.2020.25102 PMC7670313 33196807

[11] Sedani AE, Davis OC, Clifton SC, et al: Facilitators and barriers to implementation of lung cancer screening: A framework driven systematic review. JNCI J Natl Cancer Inst 114:1449-1467, 2022 10.1093/jnci/djac154 PMC9664175 35993616

[12] Stuart EA, Rhodes A: Generalizing treatment effect estimates from sample to population: A case study in the difficulties of finding sufficient data. Eval Rev 41:357-388, 2017 10.1177/0193841X16660663 PMC8317069 27491758

[13] Dahabreh IJ, Haneuse SJPA, Robins JM, et al: Study designs for extending causal inferences from a randomized trial to a target population. Am J Epidemiol 190:1632-1642, 2021 10.1093/aje/kwaa270 PMC8536837 33324969

[14] Westreich D, Edwards JK, Lesko CR, et al: Transportability of trial results using inverse odds of sampling weights. Am J Epidemiol 186:1010-1014, 2017 10.1093/aje/kwx164 PMC5860052 28535275

[15] Bareinboim E, Pearl J: A general algorithm for deciding transportability of experimental results. J Causal Inference 1:107-134, 2013

[16] Inoue K, Hsu W, Arah OA, et al: Generalizability and transportability of the national lung screening trial data: Extending trial results to different populations. Cancer Epidemiol Biomarkers Prev 30:2227-2234, 2021 10.1158/1055-9965.EPI-21-0585 PMC8643314 34548326

[17] Prosper AE, Inoue K, Brown K, et al: Association of inclusion of more Black individuals in lung cancer screening with reduced mortality. JAMA Netw Open 4:e2119629, 2021 10.1001/jamanetworkopen.2021.19629 PMC8385597 34427681

[18] Robertson SE, Joyce NR, Steingrimsson JA, et al: Comparing lung cancer screening strategies in a nationally representative US population using transportability methods for the national lung cancer screening trial. JAMA Netw Open 7:e2346295, 2024 10.1001/jamanetworkopen.2023.46295 PMC10828917 38289605

[19] National Lung Screening Trial Research Team, Aberle DR, Berg CD, et al: The national lung screening trial: Overview and study design. Radiology 258:243-253, 2011 10.1148/radiol.10091808 PMC3009383 21045183

[20] Begin a new NLST project—Learn—NLST—The cancer data access system https://cdas.cancer.gov/learn/nlst/instructions/

[21] CDC: About BRFSS: https://www.cdc.gov/brfss/about/index.htm

[22] CDC: BRFSS annual survey data https://www.cdc.gov/brfss/annual_data/annual_data.htm

[23] Dahabreh IJ, Robertson SE, Steingrimsson JA, et al: Extending inferences from a randomized trial to a new target population. Stat Med 39:1999-2014, 2020 10.1002/sim.8426 32253789

[24] Schmid I, Rudolph KE, Nguyen TQ, et al: Comparing the performance of statistical methods that generalize effect estimates from randomized controlled trials to much larger target populations. Commun Stat Simul Comput 51:4326-4348, 2020 10.1080/03610918.2020.1741621 PMC9678349 36419543

[25] Webster-Clark M, Breskin A: Directed acyclic graphs, effect measure modification, and generalizability. Am J Epidemiol 190:322-327, 2021 10.1093/aje/kwaa185 32840557

[26] Pinsky PF, Gierada DS, Black W, et al: Performance of lung-RADS in the national lung screening trial: A retrospective assessment. Ann Intern Med 162:485-491, 2015 10.7326/M14-2086 PMC4705835 25664444

[27] Kovalchik SA, Tammemagi M, Berg CD, et al: Targeting of low-dose CT screening according to the risk of lung-cancer death. N Engl J Med 369:245-254, 2013 10.1056/NEJMoa1301851 PMC3783654 23863051

[28] CDC: Weighting the BRFSS Data, 2020

[29] Ackerman B, Lesko CR, Siddique J, et al: Generalizing randomized trial findings to a Target Population using Complex Survey population data. Stat Med 40:1101-1120, 2021 10.1002/sim.8822 PMC8034867 33241607

[30] Webster-Clark M, Keil AP: How choice of effect measure influences minimally sufficient adjustment sets for external validity. Am J Epidemiol 192:1148-1154, 2023 10.1093/aje/kwad041 36813295

[31] Lund JL, Webster-Clark MA, Westreich D, et al: Visualizing external validity: Graphical displays to inform the extension of treatment effects from trials to clinical practice. Epidemiology 35:241-251, 2024 10.1097/EDE.0000000000001694 PMC10826920 38290143

[32] Austin PC: Balance diagnostics for comparing the distribution of baseline covariates between treatment groups in propensity-score matched samples. Stat Med 28:3083-3107, 2009 10.1002/sim.3697 PMC3472075 19757444

[33] Sakoda LC, Rivera MP, Zhang J, et al: Patterns and factors associated with adherence to lung cancer screening in diverse practice settings. JAMA Netw Open 4:e218559, 2021 10.1001/jamanetworkopen.2021.8559 PMC8087957 33929519

[34] Jensen T, Chin J, Baldwin J, et al: NCA—Screening for Lung Cancer with Low Dose Computed Tomography (LDCT) (CAG-00439R)—Decision Memo, 2022. https://www.cms.gov/medicare-coverage-database/view/ncacal-decision-memo.aspx?proposed=N&ncaid=304

[35] Rendle KA, Saia CA, Vachani A, et al: Rates of downstream procedures and complications associated with lung cancer screening in routine clinical practice: A retrospective cohort study. Ann Intern Med 177:18-28, 2024 10.7326/M23-0653 PMC11111256 38163370

[36] Lin Y, Liang LJ, Ding R, et al: Factors associated with nonadherence to lung cancer screening across multiple screening time points. JAMA Netw Open 6:E2315250, 2023 10.1001/jamanetworkopen.2023.15250 PMC10214032 37227725

