# Supplementary material for: Transporting Lung Cancer Screening Effects From National Lung Screening Trial to a US Screening-Eligible Population
Source: JCO Clin Cancer Inform. 2026 Oct 26;10(4):e2500280. doi: 10.1200/CCI-25-00280 (PMC13722428; doi:10.1200/CCI-25-00280)
Supplement: Supplementary file 1 [file cci-10-e2500280-s001.pdf]

Supplemental Materials

1. NLST Data Adjustments

Data Version Reconciliation

The NLST data used in our analysis had more complete follow-up than the dataset used for the primary NLST results paper (referred to as the primary NLST paper)[1], leading to slightly higher numbers of events and longer follow-up times. Consequently, the NCI team provided instructions on adjusting the event counts and person-years of follow-up to align our results with those from the primary NLST paper[2] (**Table S1**). They also supplied the expected values for lung cancer incidence, lung cancer mortality, and all-cause mortality after applying the necessary adjustments[2] (**Table S1**). Notably, these values are similar but not identical to those presented in the primary NLST paper (**Table S2**). Therefore, to compare our transported results with the original published results, we first made the recommended adjustments to our NLST dataset and then verified that our adjusted numbers matched the expected values provided by NCI before continuing with the rest of our analysis.

Table S1. Adjustments made to event definition and person-time for results comparable to primary NLST paper. Recreated from the NLST user guide provided by NCI[2].

Event type (and cutoff date)	Adjustment to event definition	Adjustment to follow-up
Lung Cancer Mortality (1/15/09)	Restrict death events to only those which occurred before 1/15/09 by using the condition DEATHCUTOFF=1	Subtract from follow-up days 94.6020 days for the LDCT arm and 392.1746 days for the CXR arm
All-Cause Mortality (12/31/09)	None	Subtract from follow-up days 58.1772 for the LDCT arm and 58.7590 for the CXR arm

Table S2. Comparison of values from the primary NLST paper with values obtained using the NCI-available NLST dataset with appropriate adjustments

Event type (and cutoff date)	Measure type	Study arm	Published numbers	Numbers from datasets

Lung Cancer Mortality (1/15/09)	Count	LDCT	356	359
Lung Cancer Mortality (1/15/09)	Count	CXR	443	448
Lung Cancer Mortality (1/15/09)	*Rate	LDCT	247	249
Lung Cancer Mortality (1/15/09)	*Rate	CXR	309	312
All-Cause Mortality (12/31/09)	Count	LDCT	1877	1904
All-Cause Mortality (12/31/09)	Count	CXR	2000	2025
All-Cause Mortality (12/31/09)	*Rate	LDCT	1121	1137
All-Cause Mortality (12/31/09)	*Rate	CXR	1202	1217

*per 100,000 person years

** Reproduced from Table 6 in the NLST data user guide provided by NCI, selecting rows with measures that were included in our analysis[2]

Positive Screen Definition

When defining a positive screen, we opted to use the American College of Radiology Lung Imaging Reporting and Data System (Lung-RADS) criteria instead of the criteria used in the NLST. This choice ensures our results align with current clinical practice.

Lung-RADS was released in 2014 by the American College of Radiology to standardize the definition of a positive screening result and guidelines for nodule management based on lung cancer risk [3,4]. A Lung-RADS category of 1 or 2 are often considered negative results and require individuals only return for their next annual screen. A Lung-RADS category of 3 or 4 are often considered positive results that require individuals to return at a shorter interval for diagnostic examination[3,4]. Applying Lung-RADS to NLST reduced the false positive rate at the baseline screen from 26.6% in NLST to 12.8% (95% CI: 12.4-13.2) and at subsequent screenings from 21.8% in NLST to 5.3% (95%CI: 5.1-5.5) [3].

Since we applied a different criterion in defining positive screen, we cannot directly compare the percentages of false-positive screens to those reported in the primary NLST paper. Therefore, we calculated the false-positive outcomes in the NLST population and used Lung-RADS to define a positive screen for comparison to our transport results. We expected the percentage of false-positive screens to be lower, as Lung-RADS has been shown to reduce false-positive results in NLST[5]. On the other hand, we were able to compare our results in false-positive rate to those reported by Pinsky while applying Lung-RADS retrospectively to the NLST results (12.8, 5.4, 5.2% in the first, second, and third round of screening)[5].

2. *Target Population Cohort Selection Flow Chart*

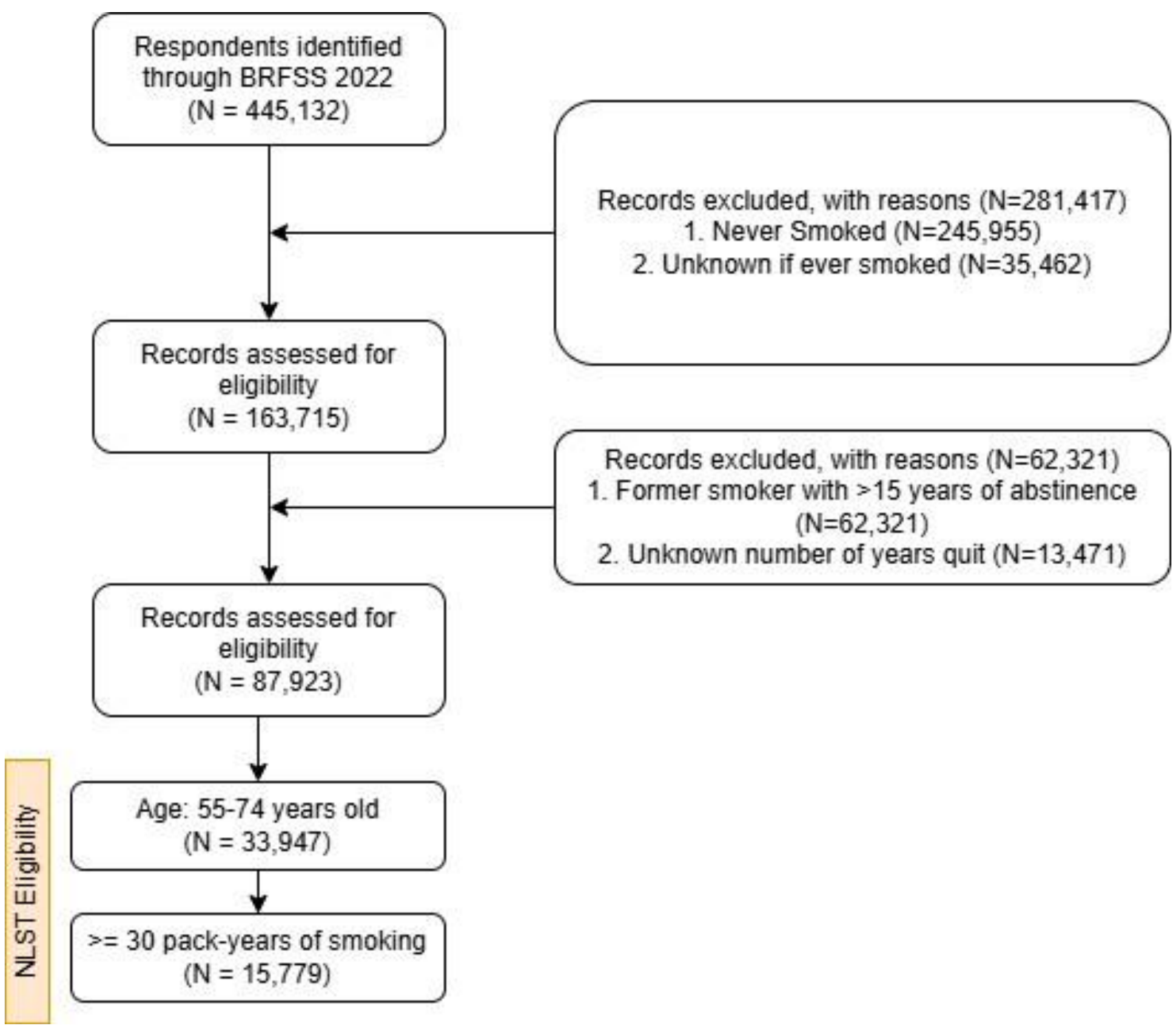

Figure S1. Flow chart depicting the process of applying the NLST eligibility criteria to BRFSS respondents to get to our target screening-eligible population

3. External Validity Assumptions

Two key assumptions that need to be fulfilled for the external validity of our transport analysis to hold are external exchangeability and external positivity. The first assumption required conditional exchangeability over trial participation, meaning that the treatment effect is independent of trial participation conditioning on the set of EMMs. We cannot empirically test whether this assumption is met but selected the set of EMMs in our IOW model by constructing a directed acyclic graph (DAG) of the relationship between exposure and outcome based on prior research and clinical input[6].

The assumption of external positivity required the probability of being sampled into the trial to be nonzero over all combinations of baseline covariates that achieve conditional exchangeability[7]. We checked for external positivity by graphical inspection of the distributions of the probability of being sampled into the trial for the NLST and BRFSS screening-eligible population[8].

4. Harm Outcome Definitions

We estimated the false-positive rate and the percentage of false-positive screens out of all positive screens in the LDCT arm (**Table S3**). We focused on positive screens as defined by Lung-RADS. We expect these percentages to be higher when applying Lung-RADS compared to the original trial's screening criteria, as Lung-RADS employs more stringent criteria for positive screens, thereby decreasing the denominator of these measures.

Table S3. Measures and formulas for our harm endpoints of interest

Measure	Formula
Primary: False-positive Rate	$\frac{\#false-positve\ screens}{\#true-negative+\#false-positive\ screens}$
Secondary: Percentage of false-positive screens	$\frac{\#false-positive\ screens}{\#positive\ screens}$

5. Abnormal or Missing Values

For NLST participants who formerly smoked but had missing data on the age of smoking cessation or whose reported age of smoking cessation was older than their age at trial randomization, we substituted the missing values with the median number of years since quitting, based on participants with complete data. There were a small number of NLST participants that did not meet the trial eligibility criteria but were included in the trial, we truncated their age, pack-years, and years since smoking cessation to the nearest values that met the criteria. For NLST participants with extreme pack-years of smoking and BMI, we truncated pack-years to the 95th percentile and BMI to the 99th percentile after consulting with clinical experts.

We did not find a high amount of missingness in key eligibility covariates and EMMs in the BRFSS 2022 data (< 5%). For missing race, ethnicity, and COPD, emphysema, or chronic bronchitis status, we imputed to the categories with the highest proportions, which were white race, non-Hispanic ethnicity, and no diagnosis of COPD. For BMI, we imputed to the median value. For years since smoking cessation, we imputed this value to the median years since smoking cessation among those who formerly smoked.

6. EMM Selection: VITT Plot and Marginal and Conditional EMM

We conducted sensitivity analyses where we screen for potential sets of EMMs for our trial sampling model based on two alternative approaches: Screen Variable Importance for Treatment Transport (VITT) plot and regression model assessment for marginal and conditional modification.

The first approach involves using the VITT plot to select variables likely associated with the outcome and the sampling mechanism into the trial population, after conditioning on other variables. We first constructed two separate multivariable logistic regression models, using the potential EMMs as covariates. The outcomes for these models were the probability of being "sampled" into the trial and the probability of experiencing the outcome of interest, respectively. We then plotted the conditional odds ratio of the outcome for each EMM from the two models against each other to create the VITT plot.

Variables were selected where both conditional associations differed from the null value of 1.

The second approach is to screen EMMs by assessing whether each variable is a conditional or marginal effect modifier using the following steps:

a. Assess marginal modification by fitting the regression model:
   i. $P(Y) = \beta_0 + \beta_1 \times X + \beta_2 \times M + \beta_3 \times X \times M$, where Y=outcome, X=exposure, M=potential EMM
      If the coefficient of the interaction term (β_3) is notably different from zero, then we would identify M as a marginal EMM[9]. For our case, we used the identity link since risk difference is our main effect measure of interest.
b. Assess conditional modification for variables identified as marginal EMM by fitting the regression model:
   i. $P(Y) = \beta_0 + \beta_1 \times X + \beta_2 \times M + \beta_3 \times Z + \beta_4 \times X \times M$, where Y=outcome, X=exposure, M=potential EMM, Z= other covariates that are EMM or potential EMM. If the interaction term (β_4) is notably different from zero, then M is a conditional EMM[9].
c. Since our primary estimand is the difference in lung cancer mortality, the minimal sufficient adjustment set for external validity would be the set of conditional EMMs on the risk difference scale[9].
d. Since our secondary estimand of interest include incidence rate ratios, we will include all potential EMMs identified via our DAG[9,10].

7. Sensitivity Model Selection and Specification

<u>Lung cancer mortality</u>

Using the VITT plot approach, we selected age, BMI, pack-years, ethnicity, sex, smoking status, COPD/emphysema/chronic bronchitis history as EMMs as their conditional association with lung cancer death trial sampling are further away from the null of 1 (**Fig S2**).

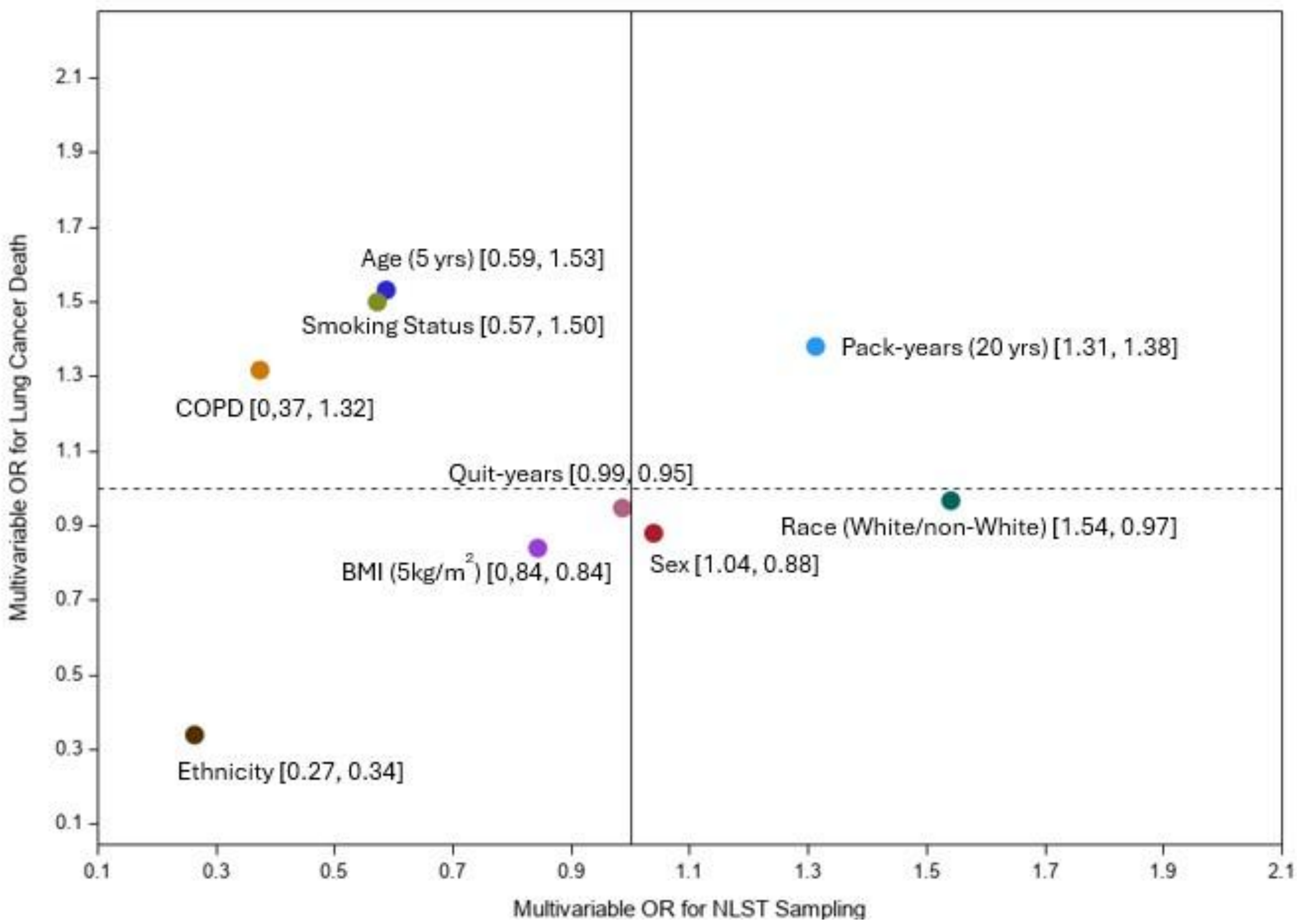

Figure S2. Variable importance for treatment transport plot used to inform variable selection for transport. This figure shows the multivariable odds ratios (specified in brackets) for each baseline patient characteristic and NLST trial sampling (x axis) lung cancer mortality (y axis).

We then additionally identify the set of conditional EMMs among the VITT plot selected variables using the regression model approach. Since it is harder to reach a traditional significance p-value of 0.05 in the linear risk model, we used the threshold of 0.5 for inclusion as an EMM and selected BMI and smoking status as conditional EMMs (**Table S4**).

Table S4. Assessments of marginal and conditional effect measure modification of lung cancer mortality by linear risk regression models. The light, grey-shaded variables under marginal effect measure modification were selected to be further assessed for conditional effect measure modification. The darker, grey-shaded variables under conditional effect measure modification were included in the IOW model.

Candidate variable	Coefficient for interaction term	P-value
**Marginal Effect Measure Modification**		
Age (5 years)	-0.00097	0.36
BMI (5 kg/m^2)	0.00156	0.05
Pack-years (20 years)	-0.00109	0.38
Race (white/non-white)	-0.00030	0.94
Ethnicity	0.00093	0.85
Sex	-0.00330	0.12
Smoking Status	0.00156	0.05
COPD/emphysema/chronic bronchitis history	0.001575	0.62
**Conditional Effect Measure Modification**		
Age (5 years)	-0.00037	0.89
BMI (5 kg/m^2)	0.00195	0.48
Pack-years (20 years)	-.00040	0.88
Sex	-0.00250	0.66
Smoking Status	-0.00414	0.45

<u>All-cause mortality</u>

Using the VITT plot approach, we selected age, pack-years, race (white/non-white), ethnicity, sex, smoking status, COPD/emphysema/chronic bronchitis history as EMMs as their conditional association with all-cause death trial sampling are further away from the null of 1 (**Fig S3**).

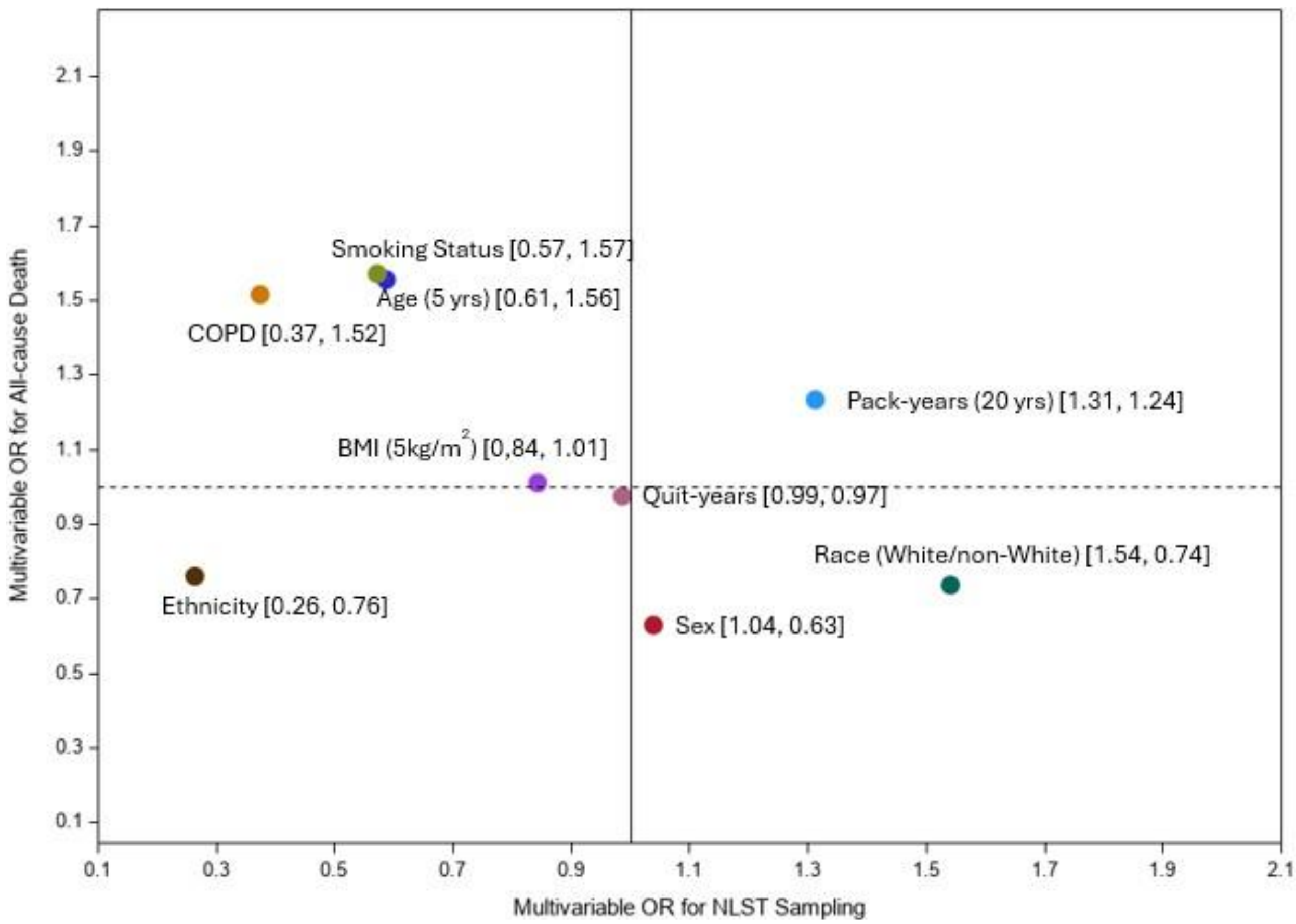

Figure S3. Variable importance for treatment transport plot used to inform variable selection for transport. This figure shows the multivariable odds ratios (specified in brackets) for each baseline patient characteristic and NLST trial sampling (x axis) all-cause mortality (y axis).

We then additionally identify the set of conditional EMMs among the VITT plot selected variables using the regression model approach. Since it is harder to reach a traditional significance p-value of 0.05 in the linear risk model, we used the threshold of 0.5 for inclusion as an EMM and selected age and race as conditional EMMs (**Table S5**)

Table S5. Assessments of marginal and conditional effect measure modification of all-cause mortality by linear risk regression models. The light, grey-shaded variables under marginal effect measure modification were selected to be further assessed for conditional effect measure modification. The darker, grey-shaded variables under conditional effect measure modification were included in the IOW model.

Candidate variable	Coefficient for interaction term	P-value
**Marginal Effect Measure Modification**		
Age (5 years)	-0.00303	0.17
Pack-years (20 years)	0.00031	0.71
Race (white/non-white)	-0.01231	0.16
Ethnicity	-0.01798	0.25
Sex	0.00146	0.74
Smoking Status	0.00016	0.97
COPD/emphysema/chronic bronchitis history	0.009567	0.16
**Conditional Effect Measure Modification**		
Age (5 years)	-0.00265	0.40
Race (white/non-white)	-0.01221	0.29
Ethnicity	-0.00315	0.91
COPD/emphysema/chronic bronchitis history	.001723	0.81

False-positive rate

Using the VITT plot approach, we selected age, pack-years, race (white/non-white), ethnicity, sex, smoking status, COPD/emphysema/chronic bronchitis history as EMMs for the main harm outcome of false-positive rate (**Figs S4-6**).

We excluded years since smoking cessation, as it did not appear to modify the false-positive status across all three rounds of screening.

As we were only focusing on the LDCT arm for the harm outcomes, we could not directly assess EMM with no different levels of exposure.

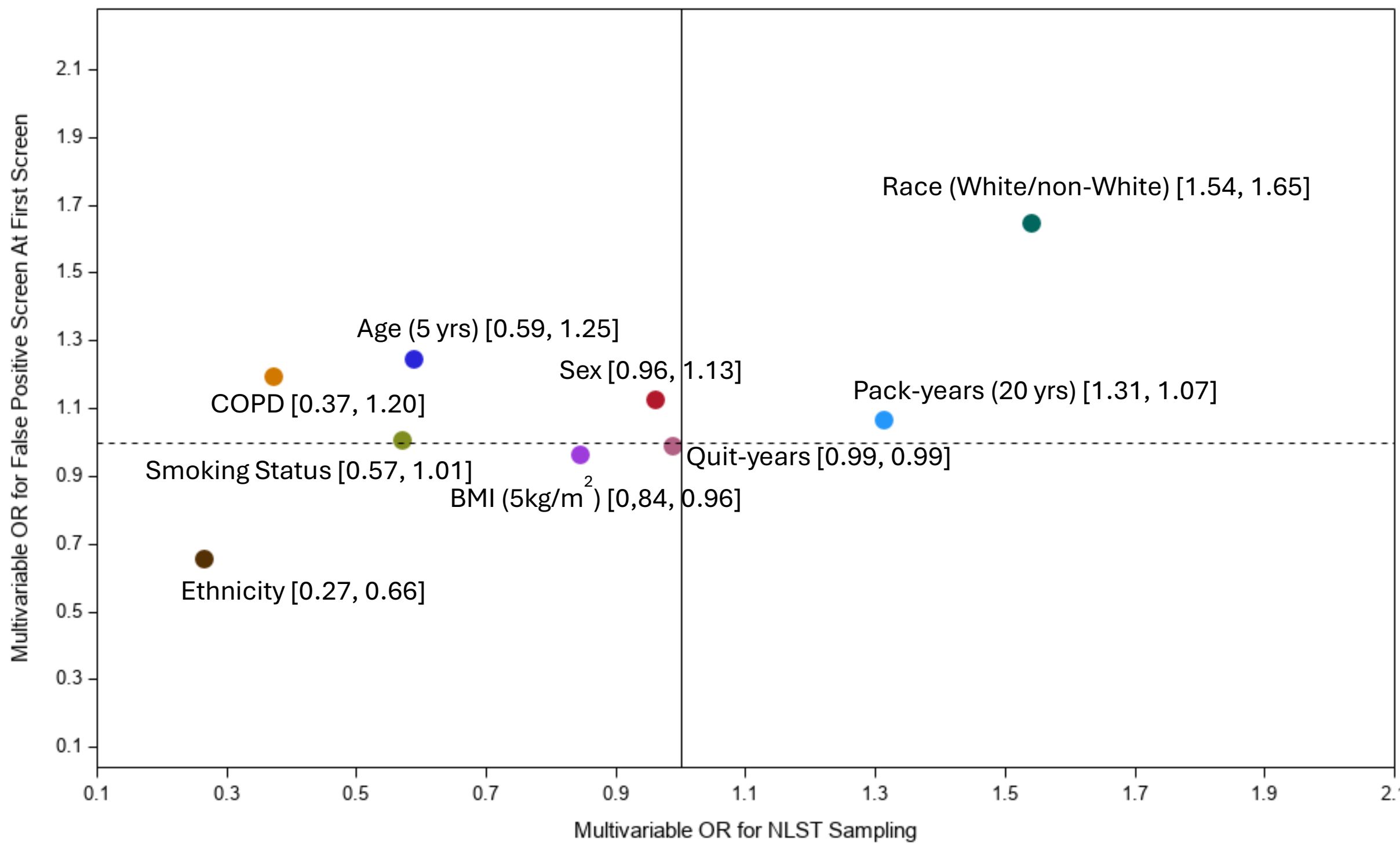

Figure S4. Variable importance for treatment transport plot used to inform variable selection for transport. This figure shows the multivariable odds ratios (specified in brackets) for each baseline patient characteristic and NLST trial sampling (x axis) false-positive result at first screen (y axis).

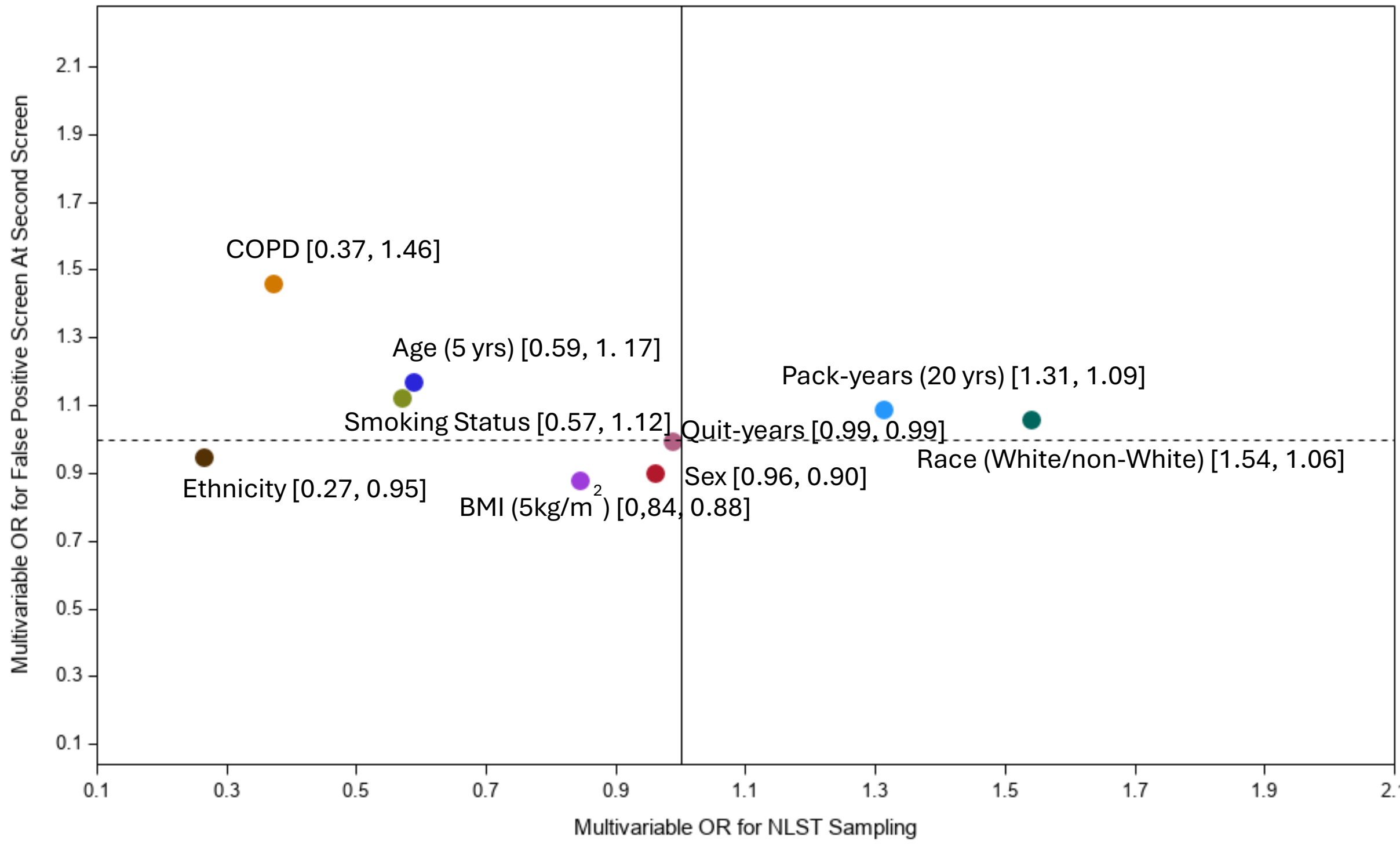

Figure S5. Variable importance for treatment transport plot used to inform variable selection for transport. This figure shows the multivariable odds ratios (specified in brackets) for each baseline patient characteristic and NLST trial sampling (x axis) false-positive result at second screen (y axis).

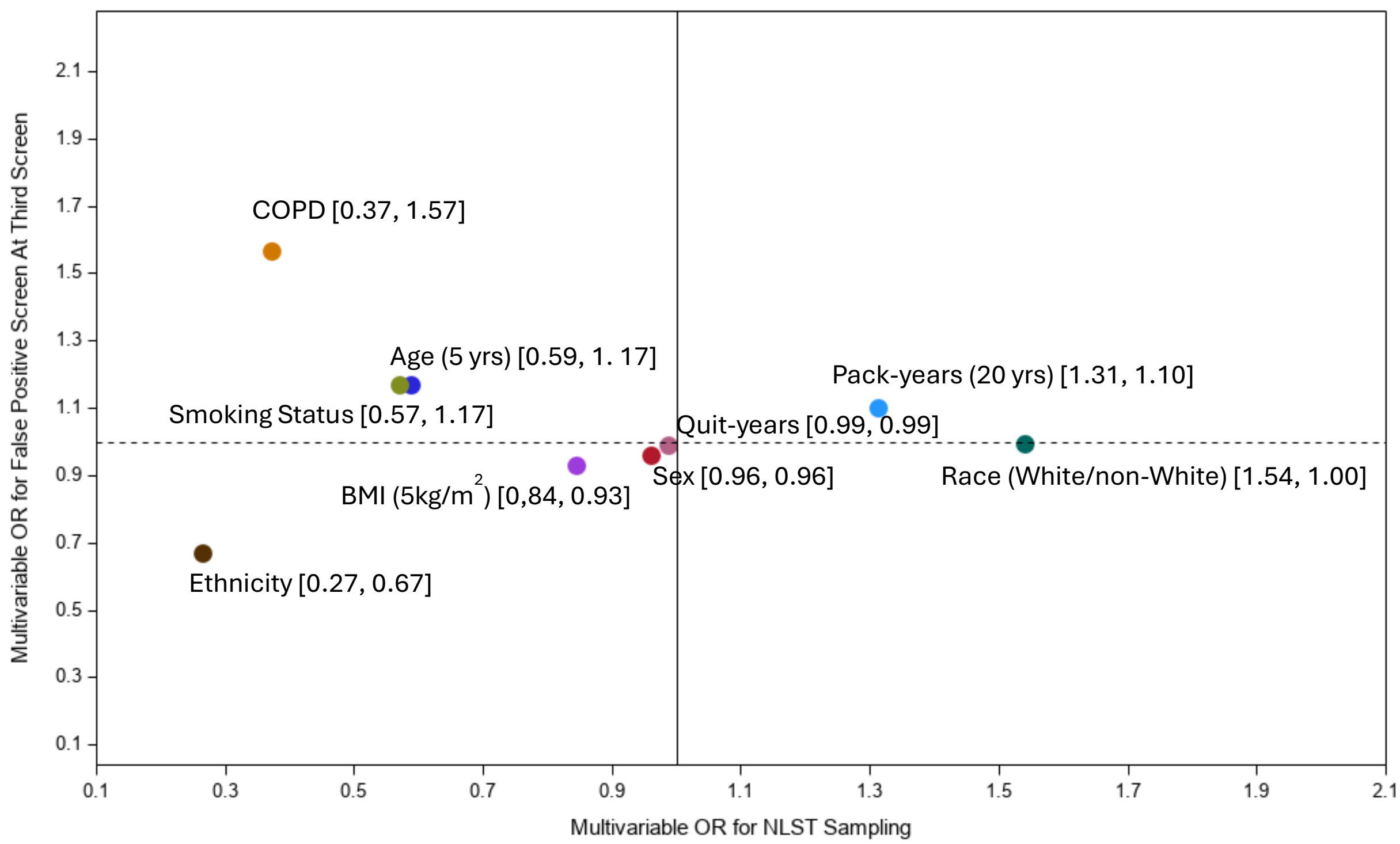

Figure S6. Variable importance for treatment transport plot used to inform variable selection for transport. This figure shows the multivariable odds ratios (specified in brackets) for each baseline patient characteristic and NLST trial sampling (x axis) false-positive result at third screen (y axis).

Table S6. Covariate Specification for Sensitivity Analysis Models

Model	Covariates Included	Interactions Included
Benefit model: Lung cancer mortality **VITT**	Age, BMI, pack-years, ethnicity, sex, smoking status, COPD/emphysema/chronic bronchitis history	Ethnicity and smoking status, ethnicity and COPD/emphysema/chronic bronchitis history, sex and smoking status, smoking status and COPD/emphysema/chronic bronchitis, age and smoking status, age and COPD/emphysema/chronic bronchitis history, and age and pack-years
Benefit model: All-cause mortality	Age, BMI, pack-years, race (white/non-white), ethnicity, sex, smoking status,	Race and sex, race and ethnicity, race and smoking status, race and COPD/emphysema/chronic bronchitis

**VITT**	COPD/emphysema/chronic bronchitis history	history, race and pack-years, ethnicity and smoking status, ethnicity and COPD/emphysema/chronic bronchitis history, sex and smoking status, sex and COPD/emphysema/chronic bronchitis history, sex and pack-years, age and smoking status, smoking status and COPD/emphysema/chronic bronchitis history, age and COPD/emphysema/chronic bronchitis history, and age and pack-years
Benefit model: Lung cancer mortality **Regression**	BMI, smoking status,	BMI and smoking status
Benefit model: All-cause mortality **Regression**	Age, race(white/non-white)	Age and race(white/non-white)
Harm model: False-positive rate **VITT**	Age, BMI, pack-years, race (white/non-white), ethnicity, sex, smoking status, COPD/emphysema/chronic bronchitis history	Race and sex, race and COPD/emphysema/chronic bronchitis history, ethnicity and sex, ethnicity and COPD/emphysema/chronic bronchitis history, age and smoking status, age and pack-years, and age and smoking status

* Age, BMI, and pack-years were specified as restricted cubic spline with three knots

8. Characteristics of the NLST, unweighted BRFSS, and survey-weighted BRFSS screening-eligible populations

Table S7. Characteristics of the NLST, unweighted BRFSS, and survey-weighted BRFSS screening-eligible populations

Characteristics		NLST<br>N = 53,452<br>N (%)		Unweighted BRFSS<br>N = 15,779<br>N (%)		Survey-weighted BRFSS*<br>N = 7,265,314<br>N (%)	
Age category (years)							
	<50	2	0.0	NA		NA	
	50-54	3	0.0	NA		NA	
	55-59	22,860	42.8	3,383	21.4	1,738,645	23.9
	60-64	16,369	30.6	4,491	28.5	2,302,867	31.7
	65-69	9,517	17.8	4,404	29.1	1,799,859	24.8
	70-74	4,697	8.8	3,501	22.2	1,423,943	19.6
	75-79	4	0.0	NA		NA	
Age continuous (years)							
	Median (IQR)	60	(57, 65)	65	(60 ,69)	63	(59, 68)
Race							
	White	48,549	91.5	13,915	90.7	6,170,529	87.7
	Black	2,376	4.5	704	4.6	532,513	7.6
	Asian	1,095	2.1	105	0.7	901,64	1.3
	Other	1,062	2.0	619	4.0	240,688	3.4
	Missing	370		436		231,420	
Ethnicity							
	Hispanic	935	1.8	496	3.2	407,990	5.7
	Non-Hispanic	52,118	98.2	15,153	96.8	6,800,498	94.3
	Missing	399		130		56,826	
Sex							
	Male	31,530	59.0	8,825	55.9	4,202,227	57.8
	Female	21,922	41.0	6,954	44.1	3,063,087	42.2

Smoking Status							
	Current	25,760	48.2	8,895	56.4	4,052,602	55.8
	Former	27,692	51.8	6,884	43.6	3,212,712	44.2
Pack-Years category							
	<20	2	0.0	NA		NA	
	20-29	18	0.0	NA		NA	
	30-39	13,719	21.8	4,235	26.8	1,967,800	27.1
	40+	39,712	78.2	11,544	73.2	5,297,514	72.9
Pack-year continuous							
	Median (IQR)	48	(39, 66)	46	(39,57)	46.00	(38, 56)
Time Since Quit							
	Current smoker	25,760	48.5	8,895	56.4	4,052,602	55.8
	≤5 years	11,338	21.3	2,403	15.2	1,144,285	15.8
	6-10 years	7,764	14.6	2,260	14.3	1,062,394	14.6
	11-15 years	7,963	15.0	2,221	14.1	1,006,033	13.9
	15+ years	307	0.6	NA		NA	
	Missing	320	0.6				
Time Since Quit							
	Median (IQR)	7	(3, 11)	8	(4, 12)	7.00	(4, 11)
BMI continuous							
	Median (IQR)	27.3	(24.4, 30.5)	27.96	(24.3, 32.3)	27.98	(24.3, 32.3)
Education							
	< High School	3,249	6.1	1,693	10.8	1,292,279	17.8
	High School/GED	12,712	23.8	5,764	36.6	2,637,082	36.4
	Some College	19,711	36.9	5,300	33.7	2,449,231	33.8
	College Grad	16,546	31.0	2,985	19.0	872,716	11.0

Missing	1,234		37		14,007	
COPD or Emphysema or Chronic Bronchitis						
Yes	9,326	17.6	5,866	37.5	2,638,174	36.6
No	43,758	82.4	9,794	62.5	4,577,847	63.4
Missing	368		119		49,293	

* We incorporated the BRFSS survey weights to reweigh the sample back to the general US population. BRFSS survey weights adjust for noncoverage, nonresponse, and demographic differences between those sampled by BRFSS and the total state population[11].

** These numbers were recorded prior to truncation and imputation that are detailed in Supplemental Material Section 5.

9. *Primary Benefit IOW Model Diagnostics*

Most covariates were well-balanced after applying IOW, with standardized mean differences (SMDs) between -0.1 and 0.1 (**Fig. S7**). Age showed moderate balance, with an SMD of 0.11. The ASAMD was 0.05, also indicating good balance across all covariates after weighting. External positivity was confirmed through graphical diagnostics (**Fig S8**). We truncated the inverse odds weights at the 99th percentile (weight=4.99) to address extreme values (**Figure S9**), as done previously[12].

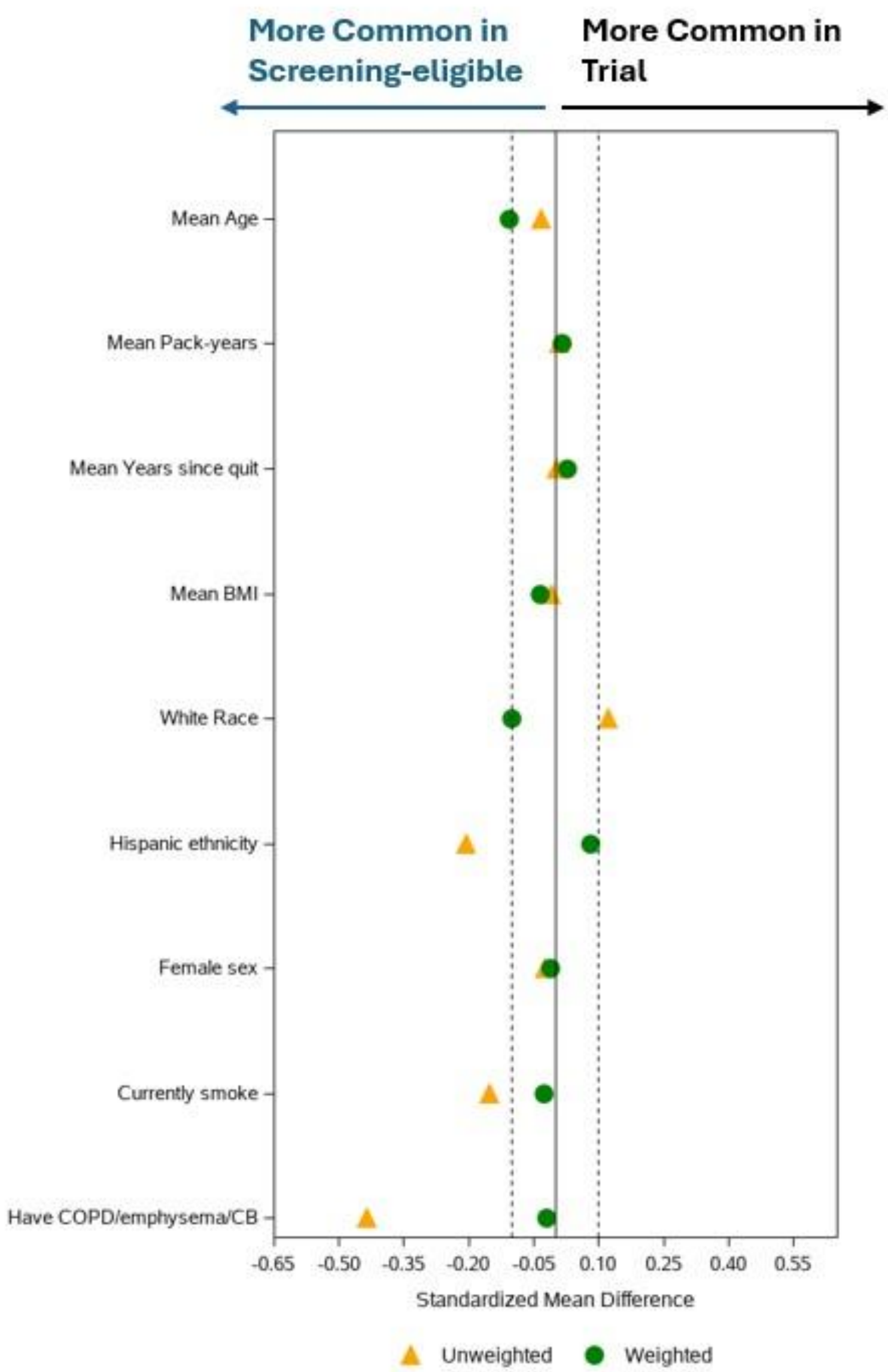

Figure S7. Covariate balance pre- (yellow triangle) and post- (green circle) IOW weighting using the fully adjusted model. SMD > 0 indicates that the trait is more common, or the mean value is greater in the NLST trial than in the BRFSS screening-eligible population, and vice-versa.

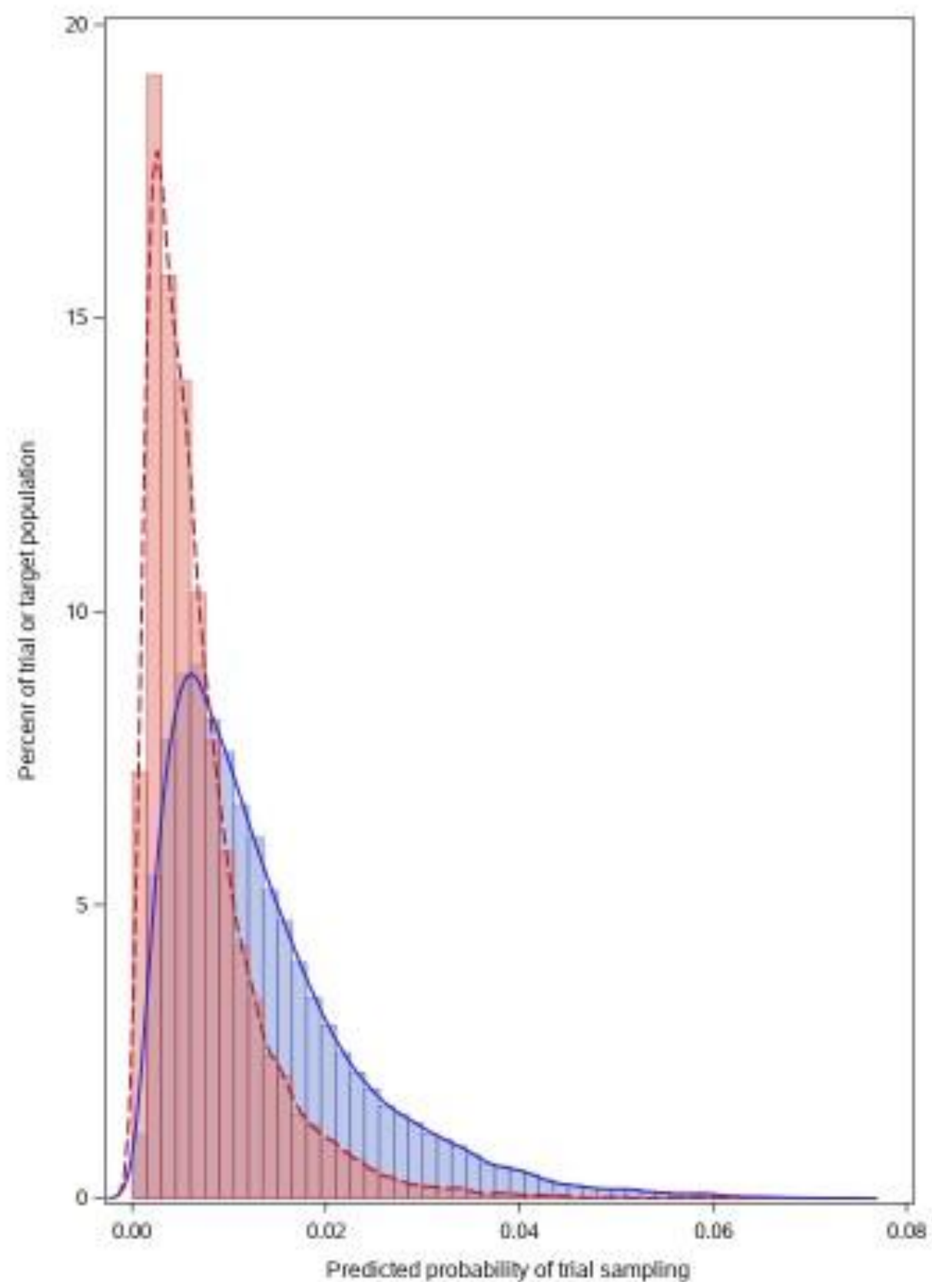

Figure S8. Distribution of predicted probability of trial sampling from the main IOW logistic regression model for NLST (blue bars) and the BRFSS screening-eligible (red bars). This is a diagnostic of positivity (i.e. whether the probability of being sampled into the trial in each EMMs stratification is non-zero).

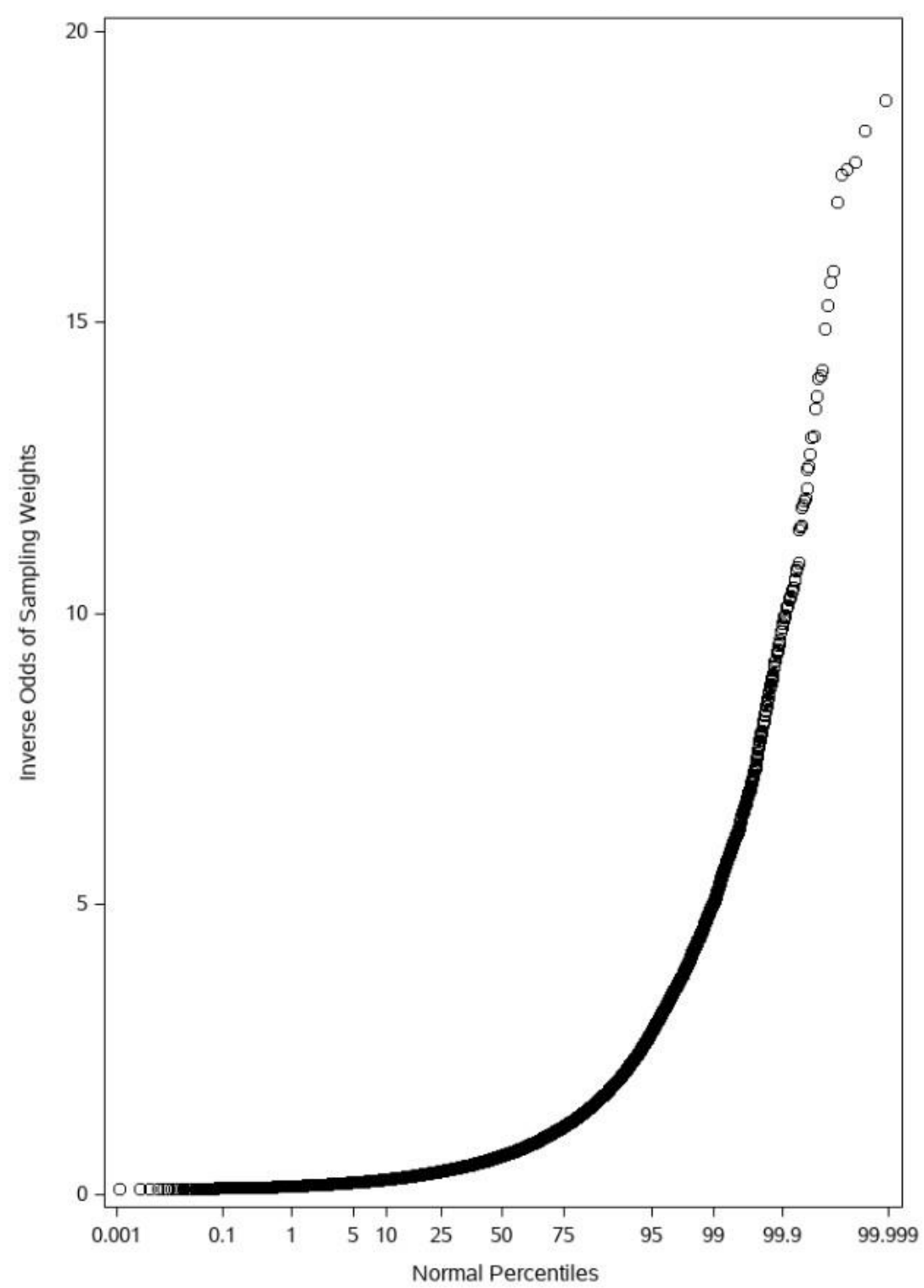

Figure S9. Distribution of the stabilized inverse odds weights. Truncation applied at the 99th percentile (weight=4.99) for analysis

10. Sensitivity Analysis Model Results: Cumulative Incidence of Mortality, Mortality Rates

Table S8. Cumulative incidence of five-year lung cancer and all-cause mortality in deaths per 10,000 screened in the unweighted and weighted trial populations.

	LDCT	CXR	Risk Difference	Risk Ratio
		Lung Cancer Mortality		
Unweighted Trial	118 (110, 128)	142 (128, 156)	-23 (-44, -3)	0.84 (0.74, 0.94)
Weighted Trial				
VITT	150 (136, 166)	179 (162, 198)	-30 (-54, -4)	0.84 (0.72, 0.97)
Conditional EMM	122 (112, 133)	143 (131, 156)	-21 (-38, -4)	0.85 (0.73, 0.99)
		All-cause Mortality		
Unweighted Trial	477 (457, 497)	513 (492, 535)	-37 (-66, -7)	0.93 (0.87, 0.98)
Weighted Trial				
VITT	626 (566, 692)	679 (642, 718)	-53 (-104, -1)	0.92 (0.85, 1.00)
Conditional EMM	587 (530, 651)	638 (609, 669)	-51 (-92, -10)	0.92 (0.86, 0.98)

Table S9. Lung cancer and all-cause mortality rates by treatment arm and population in deaths per 100,000 person years

Population	LDCT	CXR	Rate difference	Rate Ratio
		Lung Cancer Mortality		
Unweighted Trial	249 (230, 270)	312 (285, 342)	-63 (-106, -20)	0.80 (0.70, 0.91)
Weighted Trial				
VITT	320 (289, 354)	380 (346, 417)	-60 (-111, -9)	0.83 (0.73, 0.97)
Conditional EMM	260 (238, 384)	320 (298, 344)	-60 (-94, -27)	0.80 (0.72, 0.91)
		All-cause Mortality		

Unweighted Trial	1,137 (1,097, 1,178)	1,217 (1,178, 1,257)	-80 (-136, -23)	0.93 (0.89, 0.98)
Weighted Trial				
VITT	1,410 (1344, 1479)	1,520 (1455, 1588)	-110 (-212, -8)	0.93 (0.87, 0.99)
Regression	1,520 (1458, 1585)	1,610 (1550, 1672)	-90 (-171, -9)	0.95 (0.89, 1.00)

11. Secondary Benefit Outcome Results: Percentage of Early-stage Lung Cancer Diagnosis

Table S10. Percentage of early-stage cancer diagnosis among all cancer diagnosis

Treatment Arm	First Screen	Second Screen	Third Screen
	Unweighted Trial Population		
LDCT	51.9 (46.8, 57.6)	56.5 (50.0, 63.8)	58.7 (52.8, 65.3)
CXR	33.7 (28.4, 39.9)	30.1 (23.9, 37.9)	31.7 (25.4, 39.5)
	Weighted Trial Population (Main Model)		
LDCT	51.4 (44.5, 59.4)	54.5 (46.1, 64.4)	56.3 (48.3, 65.7)
CXR	35.8 (30.2, 42.4)	37.0 (29.4, 46.6)	37.9 (30.4, 47.2)
	Weighted Trial Population (VITT Model)		
LDCT	51.2 (44.1, 62.7)	52.7 (44.3, 62.7)	56.3 (48.3, 65.7)
CXR	35.0 (30.2, 42.4)	36.8 (29.4, 46.6)	38.6 (30.4, 47.2)

12. Sensitivity Analysis Model Results: False-Positive Rates

Table S11. False-positive rate of screening (%) with LDCT and using LUNG-RADS criteria to determine positive screens

Population	First Screen	Second Screen	Third Screen
Unweighted Trial (LDCT only)	12.9 (12.6, 13.2)	5.4 (5.2, 5.6)	5.2 (5.0, 5.4)

Weighted Trial (LDCT only)			
VITT model	14.2 (13.7, 14.7)	6.3 (6.0, 6.7)	5.9(5.6, 6.2)

13. Secondary Harm Outcome Results: Percentage of False-Positive Screens

Table S12. Percentage of false positive screens among those screened with LDCT using the Lung-RADS criteria

Population	% False-positive Screens
Unweighted Trial (LDCT only)	91.3 (90.8, 91.8)
Weighted Trial (LDCT only)	
Main model	90.7 (89.9, 91.5)
VITT model	90.7 (89.9, 91.5)

References

1. The National Lung Screening Trial Research Team. Reduced Lung-Cancer Mortality with Low-Dose Computed Tomographic Screening. *N Engl J Med*. 2011;365(5):395-409. doi:10.1056/nejmoa1102873

2. NLST User Guide. National Cancer Institute Cancer Data Access System. Published 2023. https://cdas.cancer.gov/datasets/nlst/

3. Pinsky PF, Gierada DS, Black W, et al. Performance of Lung-RADS in the National Lung Screening Trial. *https://doi-org.libproxy.lib.unc.edu/107326/M14-2086*. 2015;162(7):485-491. doi:10.7326/M14-2086

4. Lung Rads | American College of Radiology. Accessed December 1, 2023. https://www.acr.org/Clinical-Resources/Reporting-and-Data-Systems/Lung-Rads

5. Pinsky PF, Gierada DS, Black W, et al. Performance of lung-RADS in the national lung screening trial: A retrospective assessment. *Ann Intern Med*. 2015;162(7):485-491. doi:10.7326/M14-2086

6. Kovalchik SA, Tammemagi M, Berg CD, et al. Targeting of low-dose CT screening according to the risk of lung-cancer death. *N Engl J Med*. 2013;369(3):245-254. doi:10.1056/NEJMOA1301851

7. Dahabreh IJ, Robertson SE, Steingrimsson JA, Stuart EA, Hernán MA. Extending inferences from a randomized trial to a new target population. *Stat Med*. 2020;39(14):1999-2014. doi:10.1002/SIM.8426

8. Lund JL, Webster-Clark M, Westreich D, et al. Visualizing External Validty: Graphical Displays to Inform the Extension of Treatment Effects from Trials to Clinical Practice. *Epidemiology*. Published online 2023.

9. Webster-Clark M, Keil AP. How Choice of Effect Measure Influences Minimally Sufficient Adjustment Sets for External Validity. *Am J Epidemiol*. 2023;192(7). doi:10.1093/AJE/KWAD041

10. Webster-Clark M, Breskin A. Directed Acyclic Graphs, Effect Measure Modification, and Generalizability. *Am J Epidemiol*. 2021;190(2):322-327.

doi:10.1093/AJE/KWAA185

11. CDC. Weighting the BRFSS Data. Published online 2020.

12. Robertson SE, Joyce NR, Steingrimsson JA, et al. Comparing Lung Cancer Screening Strategies in a Nationally Representative US Population Using Transportability Methods for the National Lung Cancer Screening Trial. *JAMA Netw open*. 2024;7(1):e2346295. doi:10.1001/JAMANETWORKOPEN.2023.46295
